# Are patients receiving specialist palliative care thirsty? A qualitative interview study

**DOI:** 10.1186/s12904-026-02331-6

**Published:** 2026-09-18

**Authors:** Caroline Lythell, Anne Söderlund Schaller, Tiny Jaarsma, Maria Friedrichsen

**Affiliations:** 1 Department of Health, Medicine and Caring Sciences, Linköping University, Linköping, Sweden; 2 Clinic of Internal Medicine and Palliative Care, Vrinnevi Hospital, Norrköping, Region Östergötland Sweden

**Keywords:** Specialist palliative care, Thirst, Reflexive thematic design, Patient experience, Symptoms

## Abstract

**Background:**

Thirst is a potentially distressing yet under-researched symptom experienced by patients receiving specialist palliative care. Despite its clinical relevance, little is known about how patients experience thirst, or how it is assessed and managed in practice. This may affect patient comfort. This study aimed to describe how patients in specialist palliative home care experience thirst and investigate its impact on their daily lives.

**Design:**

A qualitative inductive design.

**Methods:**

Data were collected through semi-structured interviews with 24 patients. The interviews were analysed following the approach of Braun and Clarke’s reflexive thematic analysis. This study adheres to the EQUATOR guidelines by using Standards for Reporting Qualitative Research (SRQR).

**Results:**

Four main themes were identified describing patients’ experiences of thirst in palliative care: (1) the challenge of distinguishing thirst from dry mouth, where patients frequently conflated the two; (2) a persistent symptom in the shadow of others, where thirst was perceived as less significant compared to more distressing symptoms; (3) when thirst is not a priority, reflecting patients’ experiences of limited acknowledgement and understanding from healthcare professionals; and (4) always a bottle close, illustrating how patients continuously adapted their daily lives to manage thirst, often without reflecting on its impact.

**Conclusion:**

Recognising thirst is crucial for both patients and health care professionals, as it can impact the patient’s comfort and quality of life, even if its severity is often downplayed compared to other symptoms. Effective recognition and management of thirst, combined with clear communication from health care professionals, can improve patient care.

## Background

Palliative care is a holistic approach aimed at improving the quality of life for individuals living with serious, incurable illnesses. Its primary goal is to support patients in living as well as possible despite the progression of disease, while alleviating suffering through the optimal management of distressing symptoms [1]. Improving the quality of life for patients with cancer and other life-limiting conditions requires a comprehensive approach that addresses the full spectrum of symptom burden across the continuum of care [2]. This includes acknowledging and managing symptoms like thirst, which may significantly affect comfort and dignity at the end of life. Thirst is defined as a strong and distinct sensation, often arising from internal signals of dehydration. It is also influenced by environmental factors [3], prompting oral intake of fluids to prevent dehydration [4]. Guided by Kolcaba’s Comfort Theory [5], healthcare professionals are encouraged to assess and respond to physical, psychospiritual, and environmental needs, recognizing that even subtle symptoms like thirst can influence overall comfort and well-being. Applying this framework supports a person- centred approach that prioritizes comfort and quality of life in palliative care.

In the palliative care context, a symptom is understood as a subjective experience that reflects changes in an individual’s sensations, cognition, or psychosocial functioning, and may significantly impact their overall well-being [6]. Patients receiving palliative care, particularly in the advanced stages of illness, often experience multiple concurrent symptoms [7]. Traditionally, clinical attention has focused on well-established symptoms such as pain, nausea, and dyspnoea [8]. Emerging evidence suggests that thirst is a frequently experienced yet under-recognized symptom among patients in palliative care, according to a recent published study, thirst distress was found to be at moderate and high levels in palliative care patients, 48.4% had moderate, 38.9% had high, 4.5% had severe thirst distress and only 0,6% had no thirst [9]. Additionally, healthcare professionals have acknowledged encountering patients who suffer from thirst in palliative settings [10]. Despite its prevalence, thirst has received limited attention in both research and clinical practice. Questions regarding thirst and hydration frequently arise in palliative care, particularly when patients experience reduced oral intake or when artificial hydration is withheld or discontinued. Family members often express concerns about whether reduced fluid intake may cause thirst-related suffering. Nevertheless, little is known about how patients receiving palliative care experience and understand thirst. Given its potential to cause considerable distress and its association with complex medical conditions, pharmacological treatments, and reduced fluid intake due to disease progression [11], further research is needed to better understand this symptom from the patient’s perspective.

Thirst is a fundamental human sensation that, under normal circumstances, can usually be relieved through fluid intake. However, in palliative care, thirst and dry mouth are often influenced by multiple factors, including disease progression, medication use, oral conditions, and reduced functional ability. As patients lose independence and autonomy due to advancing illness [12], they may experience increasing difficulties in meeting basic needs such as drinking. This may intensify the distress associated with thirst, making it an important symptom to assess and address [9].

Understanding how patients experience thirst is essential for informing person-centred care and developing strategies to alleviate symptom burden. By exploring patients’ perspectives, healthcare professionals may gain deeper insight into the nature of thirst and identify approaches to improve comfort and preserve dignity in palliative care.

## Methods

### Design and aim

The study aimed to explore patients’ experiences of thirst in specialist palliative home care, and a qualitative descriptive design with an inductive approach was used to achieve this. This design was chosen to provide a rich, low-inference description of the phenomenon grounded in participants’ own words. A thematic analysis, as described by Braun and Clarke [13], was employed to identify and interpret patterns of meaning within the data. This analytical approach is well suited for addressing experiences and influencing factors [14], as it aims to identify and analyse patterns and themes, providing deeper insights that capture the complexity of patient experiences of thirst. The study followed the *Standards for Reporting Qualitative Research (SRQR)* in accordance with the EQUATOR network guidelines [15].

### Sampling and setting

Data were collected in specialist palliative home care in four cities in Sweden. These cities varied in population size, ranging from approximately 3,000 to 170,000 residents. Of the 24 participants, 16 were female and eight were male. The mean age was 74 years. Table 1 lists the demographic data of the 24 participating patients.

**Table 1:** Demographic data of the participants

Gender	
Female/Male	16/8
Age (year)	
Mean (Median)	74 (74)
Min-max	52-99
Illness	
Cancer/Heart disease	20/4
Marital status	
Married	9
Partner	2
Widower/widow	4/3
Divorced	2
Single	4
Stoma	4

A purposeful sampling approach was used to identify participants best suited to answer the research question [16]. For inclusion criteria, see Table 2. Patients were approached by a clinical nurse and asked whether they would consider participating in a study concerning thirst. Those who expressed interest were informed that the researcher would subsequently contact them to arrange a suitable time for the interview. Eligible participants were adults receiving palliative care who were able to participate in an interview. Patients were not excluded based on functional status, and individuals with severe functional impairment were eligible for inclusion provided that they were able to communicate their experiences and complete the interview.

**Table 2:** Inclusion criteria for participation in the study

• At least 18 years of age
• Affiliated with a specialist palliative home care unit in Sweden
• Reported experience of thirst
• Ability to speak and understand Swedish and adequately communicate with health care professionals
• Ability to participate in an interview
• Provided informed consent

In Sweden, specialist palliative home care provides comprehensive support around the clock for patients with life-limiting illnesses, aiming to improve the quality of life for both patients and their families. Funded publicly, the service ensures all patients have access to care, regardless of their financial situation. This approach allows patients to remain at home which promote dignity, comfort and control in their final stages of life [17].

Specialist palliative home care provides medical assistance and support through a multidisciplinary team, including physicians, nurses, assistant nurses, physiotherapists, occupational therapists and counsellors. This team collaborates to manage the patient’s symptoms and needs.

### Data collection

Data were collected in 2019 and 2022. Due to the COVID-19 pandemic, data collection was paused during 2020 and resumed in 2022. An interview guide was developed by the research group (Table 3) based on a review of relevant literature on thirst and symptom experiences in palliative care, as well as the clinical experience of the researchers The guide was designed to encourage open reflection and included broad, open-ended questions focusing on the experience, meaning, and management of thirst.

**Table 3:** Interview guide

1. Can you describe how your illness started?
2. How does a typical day look like for you right now?
3. Have you experienced thirst at any time in your life? Can you tell me about it?
4. Have you recently experienced thirst? Can you tell me about it?
5. How does thirst affect your everyday life right now?
6. Does your thirst or feelings of thirst prevent you from doing anything you would otherwise do?
7. When you feel thirsty, do you also experience other symptoms?
8. Do you discuss your symptoms, such as thirst, with your loved ones?
9. How do you deal with thirst? What do you do?
10. What helps best? What provides the longest relief from thirst? How long does it take before you get thirsty again?
11. Is there a difference in thirst depending on the time of day? Night/day? When do you feel thirsty the most? Why do you think that is?
12. Has anyone talked to you about your thirst needs? Why/why not do you think?
13. Has anyone informed you about what you can do to quench your thirst?
14. Is there anything else I haven’t asked about thirst that you think is important for me to know?

A pilot interview was conducted to test the clarity and flow of the interview guide. As the pilot interview generated rich and relevant data, it was included in the study, and no changes were made to the guide.

Individual semi-structured interviews were conducted using the interview guide. Most interviews were conducted in the participants’ homes (*n* = 21), while three interviews were conducted by telephone at the patient’s request due to health status or practical considerations. All interviews were audio-recorded using a digital voice recorder. The same recording procedure was used by both interviewers. The recordings were transcribed verbatim prior to analysis. To ensure the quality of telephone interviews, the same interview guide and follow-up strategies were used as in in-person interviews. During all interviews, clarification and follow-up questions such as “Please tell me more,” “Please explain,” “How?”, and “Why/why not?” were used to deepen the data. Interviews were conducted in a quiet environment, sufficient time was allocated, and the interviewer paid particular attention to tone of voice, pauses, and opportunities for clarification to enhance depth and understanding.

A total of 24 participants were included in the study. The sample size was guided by the aim of generating rich and meaningful data. Recruitment continued until the research team considered the dataset sufficiently rich and diverse to address the study aim and support in-depth thematic interpretation.

Interviews were conducted by A.S. (associate professor, specialist nurse in anaesthesia care) (*n* = 12) and C.L. (PhD student, specialist nurse in palliative care) (*n* = 12). The interviews lasted between 7 and 38 min, with a mean duration of 21 min. Fourteen interviews were transcribed verbatim by a professional transcriber, and the remaining interviews were transcribed verbatim by C.L.

All interview recordings and transcripts were stored on secure, password-protected servers. Access to the data was restricted to the research team. To ensure data integrity, transcripts were checked against the audio recordings for accuracy before analysis.

### Data analysis

The analysis followed the steps of Braun and Clarke’s reflexive thematic analysis [13].

Initially, the first author (CL) read and reread all the transcripts to gain an understanding of each patient’s experience. Following familiarisation with the data, written reflections were generated to capture key thoughts about the individual interview. The last author (MF, associate professor) repeated this process for the first seven transcripts to enable comparison of reflections. During the reading process, codes relevant to the research question were noted in the margins. Data were analysed both manually (on paper) and digitally using Microsoft Word^®^ (interview transcripts, coding). These reflections and codes were discussed with the other authors (MF; AS, associate professor; TJ, professor) multiple times to sense-check interpretations of the data [18]. This step was taken to verify that the authors had reached the desired level of the analysis. The data were then sorted by highlighting similar codes from the patients’ narratives. Through repeated engagement with the data, the development of initial codes, the use of mind mapping, and the documentation of analytic reflections following each interview, the researchers developed a deeper understanding of the data. This reflexive process enabled the identification of patterns of shared meaning, which were subsequently developed into the final themes. Different versions of the themes were discussed several times within the research group. Finally, after consensus was reached within the research group, the themes were defined and named. For an overview of the themes, see Table 4.

**Table 4:** Examples of the analysis process

Interview excerpts (interview number)	Generating initial codes of relevance to the research question	Generating themes	Defining and naming themes	Questioning and reflection process	Themes of meaning
“I: Have you experienced thirst lately? R: Yes … I must say I have, my mouth gets dry.” I.9	Mixing up thirst and dry mouth	Dry mouth is defined as thirst	Distinguishing between thirst and dry mouth	Patients confuse thirst with a dry mouth. When asked about thirst, they talk about a dry mouth.	The challenge of distinguishing thirst from dry mouth
”I asked many doctors about it (why I am thirsty). And… Yes, yes, they say. It’s the medicine. I think that’s why I’ve been so thirsty and… but it must have been something that caused it. That I’ve been so thirsty…” I.1	Wondering what causes thirst	Understanding origin of thirst	The request to understand the origin of thirst	The patients talk about wanting to understand why they are thirsty but this need for knowledge is not meet. They bring up their own ideas to why they are thirsty.	When thirst is not a priority
“I, it is as I say, that the thirst is not the worst thing, nor the dry mouth. It’s the pain and this feeling of insecurity.” I.2	Thirst is not the worst thing. It is pain and insecurity	Thirst is compared to pain and insecurity, not the worst thing	A persistent symptom in the shadows of others	This theme is about how patients discuss thirst as a persistent symptom, but pain and a sense of insecurity are the worst symptoms. So even if thirst is something that is persistent, it is manageable, compared to pain.	A persistent symptom in the shadows of others
“Of course, you have to make sure that you have some fluids nearby, especially if you are going out. You have to bring a bottle of water.” I.6	Always needing to drink	Ensuring relief	Ensuring relief from thirst	Many of the patients say that they do no longer feel that thirst impact their day, however, to handle their thirst they always make sure to have a bottle of drink close by. They also talk about restrictions (not drinking before bed to avoid toilet at night)	Always a bottle close

### Reflexivity

The first author is a specialist nurse in palliative care and acknowledges that professional background and clinical experience may influence the interpretation of patients’ expressions of thirst. While familiarity with the clinical context and symptomatology can offer valuable insight, it also necessitates ongoing reflexivity to minimise the risk of bias or assumptions based on prior knowledge. Throughout the research process, efforts were made to maintain an open and critical stance, prioritising the participants’ perspectives and carefully considering how the researcher’s understanding of palliative care might shape the analysis and interpretation of the findings.

## Results

The analysis resulted in four themes that captured patients’ experiences regarding thirst in everyday life within specialist palliative home care. The themes were: The challenge of distinguishing thirst from dry mouth; A persistent symptom in the shadows of others; When thirst is not a priority; and always a bottle close.

### The challenge of distinguishing thirst from dry mouth

This theme highlights the difficulty patients experienced in differentiating between thirst and dry mouth. When asked about thirst, several patients initially responded affirmatively but subsequently described sensations such as oral dryness, stickiness, or a lack of saliva, rather than a desire or need to drink fluids. Follow-up questions were used to clarify whether participants referred to thirst or symptoms localised to the oral cavity. Despite these efforts, patients often continued to describe experiences consistent with dry mouth rather than thirst, indicating that the distinction between these sensations was not always clear to them.

Interviewer (I): “Can you tell me something about your thirst?

*Respondent (R): “Yes*,* it’s dry in the mouth.” I.5*

Descriptions of thirst frequently began with accounts of dry mouth, suggesting an overlap or conflation between the two sensations. Patients described dry mouth as a feeling of oral dryness, including the tongue sticking to the roof of the mouth, difficulties speaking, and a perceived lack of saliva. These accounts illustrate the challenge patients faced in distinguishing between thirst and dry mouth as separate phenomena.

However, other patients responded that they could distinguish between thirst and dryness of the mouth, and they described thirst as an intense craving to drink, a dryness often accompanied by thoughts about drinking, an overwhelming desire for fluid, and an urge to drink.

*”Yes*,* so the dry mouth*,* I get very*,* very dry in the whole mouth. How can I describe it? The tongue can almost get stuck sometimes. And then in between*,* I feel thirsty. I can think of … very cold water*,* for example*,* like that…” I.10*

Patients also described difficulties in articulating what thirst felt like. Although several initially expressed uncertainties when asked to describe the sensation, many stated that they frequently experienced thirst. Thirst was often portrayed as a straightforward and familiar bodily signal that did not require further reflection. When experiencing thirst, patients reported drinking fluids as an automatic and self-evident response.

A similar pattern was observed in relation to dry mouth, where drinking water was also described as the primary strategy for relief. This suggests that both thirst and dry mouth were managed in a similar, habitual manner, without patients necessarily distinguishing between the underlying sensations.

“Well, I don’t know. I just get thirsty, quite simply, and then I drink water.” I.4

### A persistent symptom in the shadows of others

Patients viewed thirst as a minor inconvenience in the broader context of terminal illness, often minimising the issue of thirst because they felt they faced more urgent and significant problems. Patients described thirst as a troublesome symptom, but at the same time, they emphasised that it could not be compared to other symptoms. They explained that thirst was just one symptom among others, such as nausea, pain and concerns about an uncertain future due to life-limiting illness.

Patients were more focused on other aspects of their illness and life that had a more severe negative effect. Nevertheless, thirst had daily consequences in their routines, even if it was not at the forefront of their concerns.

*“My whole life is completely upside down now*,* so I can’t say that thirst is worse than*,* than everything else. It is as I say that the thirst is not the worst thing*,* nor the dry mouth. It’s the pain and this feeling of insecurity. I.2"*

Another factor contributing to the patients’ tendency to downplay the significance of thirst was that, at this stage of their illness, thirst could be temporarily quenched by drinking, even though the relief was short lived, and the symptom remained persistent and intrusive. In contrast, symptoms such as pain or feelings of uncertainty about the future were perceived as more challenging to manage, making thirst seem comparatively less significant.

*“Thirst is a marginal problem in the larger context. I.7"*

Another factor influencing the patients’ perceptions that thirst was a minor issue was their view that thirst is a natural and normal bodily function. Even though they described their sense of thirst differently from “normal thirst”, they still regarded it as something ordinary. This normalisation downplayed the distress caused by the symptom. Others described a more burdensome experience. For these patients, thirst dominated their thoughts, was persistently uncomfortable, and led to excessive fluid intake, occasionally resulting in physical discomfort such as stomach pain. The extent to which thirst occupied their daily lives was describes as drinking all the time. Nonetheless, they often claimed that thirst did not significantly affect their daily lives. This apparent contradiction may reflect a tendency to normalise or downplay the impact of thirst, despite clear indications of suffering.

*“I drink basically all the time.” I.24*

### When thirst is not a priority

Some patients expressed concern about the underlying cause of their thirst and emphasised the importance of healthcare professionals taking this issue seriously. They described actively seeking explanations yet often receiving brief and general responses attributing their thirst to medication side effects.

*” I asked many doctors about it (why I am thirsty). And… Yes*,* yes*,* they say. It’s the medicine. I think that’s why I’ve been so thirsty and… but it must have been something that caused it. That I’ve been so thirsty…” I.1*

When raising these concerns, patients felt that their experiences were not fully acknowledged by healthcare professionals. Although practical advice was offered, such as the use of mouth sprays or salivary stimulants, although these interventions were often perceived as ineffective and, at times, inappropriate for their symptoms. Patients described that such recommendations were provided without deeper engagement or genuine interest in their individual experience.

Overall, patients expressed a strong desire to be listened to and to receive a more meaningful explanation for their thirst, rather than a simplified attribution to medication side effects.

*“When I say that I*,* I feel very thirsty*,* then they should look at it*,* check it. You have to check it. I.1"*

Due to a lack of knowledge and lack of interest among healthcare professionals, the issue of the origin of thirst was overlooked. Patients felt they were left to manage their thirst on their own, relying on measures suggested by healthcare professionals that had little or no effect in relieving their symptoms.

“Yes, I’ve talked a little with the health care professionals and asked a bit. I’ve bought some products, but I don’t think it helps much. There are a few different ones in the pharmacy, but I don’t think it gives any greater results actually.” I.11

Their perception of the origin of thirst, however, was not solely influenced by what healthcare professionals told them. It was also shaped by their own observations of symptoms, which coincided with changes in their health status or treatment. Some recognised that thirst often appeared in connection with phases of deterioration, such as disease progression or oral fungus. They also identified other potential causes of thirst, such as gastrostomy, which they believed had an impact. Patients with a gastrostomy noted that their thirst began or worsened only after receiving it and theorised that the gastrostomy was the cause. They described their thirst as intense and difficult to quench.

“Normally, I haven’t been thirsty. But now, I am so thirsty that I go crazy sometimes. So I can’t get enough. I drink until I get a stomach-ache. Because I need more….” I.12

### Always a bottle close

When patients described their experiences of thirst, they often portrayed it as something that did not require much attention. At the same time, they described a continuous need to ensure access to fluids, commonly by always keeping something to drink within reach. Managing thirst appeared to be an ingrained habit, integrated into daily routines to the extent that it no longer required conscious reflection.

*“No*,* it [thirst] doesn’t really do that [disturb everyday life]. It’s part of everyday life.” I.23*

Although patients initially claimed that thirst did not significantly affect their daily lives, further reflections revealed a more complex picture. They described a persistent and unpleasant sensation of thirst that could not be fully alleviated. When access to fluids was limited, this resulted in discomfort and sometimes anxiety.

”I often want to have a water bottle with me so I can drink. And I feel thirsty all the time, and dry in the mouth. I.1”

Despite these experiences, patients tended to downplay the impact of thirst. Many explained that as their daily lives were already restricted, primarily spent at home, the constant need to drink was not perceived as a major additional burden. Instead, it was accommodated within their existing routines by ensuring that water was always nearby.

Practical adaptations were also described, such as adjusting fluid intake to manage nighttime awakenings. Although these disruptions were acknowledged as inconvenient, they were generally regarded as minor and manageable.

”Because then I try to drink quite a lot. But then it’s that [I] don’t drink too much, because then there will be toilet visits. 1.6”

## Discussion

As far as the authors are aware, this study is unique in that the phenomenon of ‘thirst’ has never been qualitatively studied in patients in palliative care. The main results of the study showed that patients tended to minimise the importance of thirst, meaning that thirst may not be the most important symptom in palliative care. At the same time, the results showed that thirst is a distressing symptom for patients, which may not be fully addressed due to several reasons, such as lack of engagement by healthcare professionals, and the confusion of thirst symptoms and symptoms of dry mouth.

The confusion meaning that patients in the study could not easily distinguish between thirst and dry mouth. This raises the question of whether they can truly differentiate between these symptoms and whether this distinction matters, given that all symptoms, regardless of severity, should be acknowledged. According to Comfort Theory, healthcare professionals play a vital role in providing comfort and alleviating distress, ensuring that patients’ physical, emotional, and psychological needs are met [5]. Through comprehensive symptom assessment, healthcare professionals can better understand the sources of discomfort and identify appropriate interventions [8]. The Revised Oral Assessment Guide (ROAG) [19] is commonly used in Sweden to assess oral health. However, it does not include thirst. This may suggest that thirst is not systematically prioritised in clinical practice. The routine use of assessment tools that include measures of thirst intensity, mucosal hydration and oral dryness could help to prevent or alleviate thirst [20]. In palliative care, inadequate management of symptoms such as thirst can lead to unnecessary suffering, highlighting the importance of addressing patients’ basic needs [21] and proper assessment of symptoms.

However, it is of important to acknowledge that findings suggest that patient self-assessment tools may not accurately reflect the actual symptom burden. One study showed that patient self-assessment tools of dry mouth were not a reliable indicator of actual oral dryness oral dryness [22], this raises important questions about the reliability of patient self-assessment in relation to thirst as well. This highlights the need for more objective and observational approaches when evaluating symptoms that are difficult to assess, especially in palliative care. One instrument developed to measure thirst is the thirst distress scale [23], The scale (first developed for renal function) [24] quantifies the degree to which a person is bothered by thirst. It may have potential for use in palliative care settings, although validation in this population is needed before its applicability can be established.

This study also revealed that patients often felt that their concerns about thirst were not properly addressed by healthcare professionals. Rather than addressing their thirst, patients often received advice about dry mouth (e.g. mouth tablets and sprays). This may indicate a mismatch between patients’ experiences of thirst and the interventions offered by healthcare professionals. This also highlights the importance of being able to differentiate between thirst and dry mouth. Whether this confusion regarding the management of symptoms is due to a lack of knowledge or just uncertainty about how to manage symptoms, it is crucial for healthcare professionals to be transparent with patients about their limitations. According to one study, healthcare professionals believed that they were aware of their limitations and only discussed symptom management with patients when they felt they had sufficient knowledge of the symptoms in question [25]. A Swedish study suggests that healthcare professionals should make themselves available to answer patients’ questions and encourage open discussion in order to reduce medical uncertainty [26]. Although the study was conducted in a different context, these findings remain relevant, emphasising the importance of accessibility and communication in building trust.

Furthermore, due to a lack of acknowledgement and differentiation by healthcare professionals, patients were forced to rely on measures that provided little or no relief. This lack of empathy and limited attention to thirst highlights a gap in the holistic care required in palliative settings. Previous research suggests that some simple interventions, such as mint-flavoured ice cubes, may relieve both thirst and dry mouth. These findings highlight the importance of assessing patients’ symptoms carefully and selecting interventions that address the specific symptom experienced, while recognizing that some interventions may benefit both conditions [27].

While patients described thirst as a bothersome and difficult symptom to manage, they frequently overshadowed by symptoms they considered to be more urgent, such as pain and death anxiety. This tendency to minimise thirst made it challenging to fully understand the true impact it had on patient wellbeing. Compared with palliative care, thirst has received greater research attention in intensive care settings. Previous studies suggest that thirst is a common and distressing symptom in ICU patients and may be associated with other symptoms [20]. However, the available evidence has limitations, and the evidence regarding thirst in palliative care remains scarce.

The results also raise questions about thirst in the final days of life, a period where patients often experience a decline in bodily function and worsening of symptom burden. Factors such as malnutrition, dysphagia and cognitive impact often impair oral intake. This may contribute to decreased quality of life. The inability to eat or drink may result in emotional distress for patients and caregivers [28, 29].

It also raises the question of the difficulty of investigating thirst during the last days of a patient’s life. Previously, healthcare professionals in specialist palliative care units have interpreted signs of thirst in terminally ill patients as sucking on the mouth stick [10]. A 1994 study [30] found that 66% of terminally ill patients experienced thirst. However, it was deemed not to be problematic as it could be alleviated with simple measures. However, this study is not particularly relevant today. Healthcare has evolved significantly, with patients now living longer with serious illnesses. Methodologically, the study only included 32 cognitively intact participants, lacing a control group and strict study protocol. Therefore, while the study offers useful insights, its findings should be interpreted with caution and in the context of more recent research.

### Implications for clinical practice

It is important to acknowledge thirst and to distinguish between the sensation of thirst and the experience of a dry mouth, as this distinction influences the choice of appropriate treatment. While oral oil may relieve the discomfort associated with a dry mouth, it does not address the underlying sensation of thirst. Understanding patient thirst in palliative care is important to improving symptom management and ensuring comprehensive care. The results of the present study highlight the need for greater awareness and understanding of thirst as a significant symptom in palliative care. For those working in palliative care, this means acknowledging thirst as an issue that can impact patients’ comfort and wellbeing, despite being overshadowed by more severe symptoms like pain or anxiety. Healthcare professionals should take a more active role in addressing thirst in palliative care, ensuring that it is neither overlooked nor underestimated. By initiating conversations about thirst and assessing patients’ ability to express or manage this symptom, clinicians can provide appropriate and timely interventions. Attention should also be given to patients who are unable to communicate or independently satisfy their thirst, to prevent unnecessary suffering during their final days, otherwise patients may die thirsty without the possibility to express it.

### Strengths and limitations

The study’s trustworthiness was ensured through measures that strengthened credibility, dependability and confirmability. A pilot study was carried out to test the data collection method, and the results were illustrated with quotations to clarify the link between the data and the analysis. The analysis process was described transparently.

Regarding the interview guide and depth of data, it could be argued that some of the interview questions were not particularly open-ended. However, this is in line with previous studies [10], which have shown that reflection about thirst is uncommon and it can be difficult for patients and healthcare professionals to discuss thirst. To encourage patients to talk freely, questions presented in the study were followed up with probing questions. We acknowledge that interviews of relatively short duration (mean 21 min) may raise questions about depth. The length of interviews reflected participants’ health status, energy levels, and tolerance, which are important ethical considerations in palliative care research.

Additionally, although participants were informed about how their data would be used, the study findings were not returned to participants in an accessible format. This decision was informed by the study context, as participants were receiving palliative care and many were unlikely to be able to engage with study outputs by the time the research was completed.

## Conclusion

In conclusion, addressing thirst is important for patients and healthcare professionals, as it affects patients’ comfort and quality of life, even if patients tend to downplay the severity of the symptom compared to other, more distressing symptoms. Proper recognition and management of thirst, along with clear communication from healthcare professionals, can enhance patient care. By acknowledging all symptoms, no matter how minor they may seem, healthcare professionals can ensure that patients are as comfortable as possible and that their quality of life is prioritised during the most challenging stages of illness.

## Data Availability

Due to the sensitive nature of the data and the need to protect participant confidentiality, the datasets of the current study are not publicly available but can be obtained from the corresponding author upon reasonable request.
